# Gene profiling and signaling pathways of *Candida albicans* keratitis

**Published:** 2008-09-26

**Authors:** Xiaoyong Yuan, Bradley M. Mitchell, Kirk R. Wilhelmus

**Affiliations:** Sid W. Richardson Ocular Microbiology Laboratory, Cullen Eye Institute, Baylor College of Medicine, Houston, TX

## Abstract

**Purpose:**

To compare the global gene expression patterns in uninfected and fungus-infected mouse corneas at the onset of *Candida albicans* keratitis.

**Methods:**

Fungal keratitis was generated by scarifying the corneal epithelium of BALB/c mice followed by topical inoculation with *Candida albicans*. Corneal infection was allowed to progress for one day, and total RNA was then extracted from excised corneas. Microarray was performed to detect 45,102 murine genes and processed to identify genetic regulation of signaling pathways. Selected genes encoding interleukins (IL), chemokine ligands, and other cytokines were confirmed by quantitative real-time reverse transcriptase polymerase chain reaction (RT–PCR).

**Results:**

Compared to mock-inoculated control eyes, genetic microarray analysis of *Candida albicans* keratitis showed that 3,977 genes (8.8%) changed at least twofold and 1,672 genes (3.7%) changed at least fourfold. Hierarchical clustering identified that upregulated genes affected immune and inflammatory responses, intercellular signaling, and cellular proliferation. Pathways having more than 20% of their genes significantly upregulated signaled leukocyte extravasation, increased interleukin production, and affected toll-like receptors. Upregulated transcript levels for IL-1β and IL-6 were confirmed by real-time RT–PCR.

**Conclusions:**

Host gene expression during the initial stage of *Candida albicans* keratitis involves pathways contributing to acute inflammation mediated by interleukins and other signals of leukocyte recruitment. This murine study confirms the involvement of innate immunity in the cornea during the initiation of *Candida albicans* keratitis.

## Introduction

Fungal keratitis is a vision-threatening disease [1]. Often due to filamentous fungi in the tropics, corneal infection due to *Candida albicans* has a worldwide distribution [2]. Although linked to environmental exposure, especially in the setting of injury or keratopathy [3], the pathogenesis of fungal keratitis is not fully understood. Fungal virulence factors allow growth and invasion into the corneal stroma [4,5], but host responses also contribute to the pathophysiology of corneal disease. Studies have recently shown that matrix metalloproteinases [6,7] and other inflammatory mediators have key roles in the progression and outcome of keratomycosis. To gain further insight into the molecular processes of keratomycosis, this study investigated the global genetic expression pattern at the onset of *Candida albicans* keratitis.

Corneal infection by *Candida albicans* triggers an inflammatory response that leads to visual loss in more than half of affected eyes despite therapy [2]. The prompt inflammatory response consists predominantly of neutrophils [8], although lymphocytes and macrophages are also involved [9]. Our studies suggest that proinflammatory mediators play important roles at the onset of *Candida albicans* keratitis, but the early molecular events that are involved in the corneal and immune responses remain unclear. Therefore, we examined the early transcriptional profile of the fungus-infected cornea in comparison to controls. Observed changes in the corneal transcriptome were categorized to suggest potentially important pathways contributing to corneal inflammation during fungal keratitis.

Gene microarray methodology has evolved as an important technique to investigate the mechanisms for many ocular disorders including microbial keratitis [10,11]. In this study, we systematically evaluated the host gene expression during the early stage of *Candida albicans* keratitis in a mouse model. To identify molecular cascades of potential importance in pathogenesis, we compared corneas of mice that developed fungal keratitis after epithelial scarification to uninfected, scarified controls. Results were validated for selected genes encoding interleukins and other cytokines.

## Methods

### Experimental fungal keratitis

A human isolate of *Candida albicans* strain SC5314 [12] was grown on Sabouraud dextrose agar (Difco, Detroit, MI) for three days at 25 °C. Yeasts were harvested and diluted in sterile phosphate-buffered saline (PBS) to yield 2×10^5^ colony-forming units (CFU)/μl [12]. Thirty adult, female BALB/c mice six- to eight-weeks old (Harlan Sprague-Dawley, Houston, TX) were anesthetized intraperitoneally with rodent combination anesthesia, and the corneas of right eyes were superficially scarified [13]. A 5 µl inoculum (1×10^6^ CFU) of *Candida albicans* was applied to the scarified cornea to induce experimental keratitis. An equivalent volume of sterile PBS diluent was applied to mock-infected controls. Animals were treated in accordance with the ARVO Statement for the Use of Animals in Ophthalmic and Vision Research, and protocols were approved by the Baylor College of Medicine Institutional Animal Care and Use Committee. The severity of keratomycosis was categorized at 24 h post-inoculation (p.i.) [13] when mice were sacrificed and right eyes enucleated.

### RNA extraction

Mouse corneas were dissected at 24 h p.i., and surrounding tissues were removed. Corneas were pooled in sets of five for total RNA extraction with triplicate groups being prepared for *Candida albicans-*infected and mock-infected control groups. RNA was extracted from each pooled sample using RNeasy MicroKit (Qiagen, Valencia, CA), and samples were treated with DNase (Qiagen) and stored at −80 °C until use.

### Microarray analysis

Microarray analysis was performed in the Microarray Core Facility of Baylor College of Medicine (Houston, TX). RNA samples with acceptable spectrophotometric ratios (OD 260/280 ratios greater than 2.0 and 260/230 ratios greater than 1.5) and good electrophoretic profiles were used for microarray analysis. Affymetrix Genechip (Affymetrix, Santa Clara, CA) protocols were applied to qualified samples for two cycles of amplification. The double-stranded cDNA end product was processed with an in vitro transcription kit (Affymetirx) to produce biotin-labeled cRNA that was quantified with a ND-1000 spectrophotometer (NanoDrop Technologies, Wilmington, DE). Labeled cRNA (15 μg) was fragmented and rechecked for concentration. A hybridization mixture containing Affymetrix spike-in controls and fragmented labeled cRNA was hybridized overnight at 45 °C with rotation at 60 rpm then washed and stained with a streptavidin and R-phycoerythrin conjugate stain on a fluidics station 450 (Affymetrix). Following signal amplification with biotinylated antistreptavidin, stained arrays were scanned and recorded using Affymetrix GCOS software version 1.4 (Affymetrix) then adjusted and analyzed with BioConductor software [14]. Pairwise comparisons were made between infected and mock-infected control groups. Raw p values were adjusted by the Benjamini-Hochberg method for 5% false discovery rate to yield adjusted p values [15]. The criteria for significance of differentially regulated genes were established as greater than or equal to a twofold change with an adjusted p value of less than or equal to 0.05. Pathways were analyzed (Ingenuity Systems, Redwood City, CA) to determine the ratio of known genes within each pathway that were significantly upregulated during *Candidas albicans* keratitis relative to the total number of known genes.

### Reverse transcription of RNA and quantitative real-time RT-PCR

Total RNA isolated from corneas one day after inoculation was spectrophotometrically quantified at 260 nm. The first-strand cDNA was synthesized from 0.4 μg of total RNA with Ready-To-Go You-Prime First-Strand Beads (GE Healthcare, Princeton, NJ) and random hexamers (Applied Biosystems, Foster City, CA). Real-time polymerase chain reaction (RT-PCR) was performed using TaqMan Assays (Table 1) and TaqMan Gene Expression Master Mix and Assays (Applied Biosystems). Primers specific for interleukins, *IL-1β*, *IL-6*, and *IL-23α*; chemokine ligands, *CCL4* and *CCL7*; chemokine receptors, *CCR3* and *CCR5*; and transforming growth factor-beta 2 (*TGF-β2*) gene transcripts (Applied Biosystems) were used to quantify gene expression levels. The threshold cycle (C_T_) for each target mRNA was normalized to glyceraldehyde-3-phosphate dehydrogenase (*GAPDH*) mRNA and averaged. Gene expression levels were calculated from normalized C_T_ results, and mean results were used to determine relative fold changes between experimental groups. Two-group comparisons were analyzed with Student’s *t*-test. A p value less than or equal to 0.05 was considered statistically significant.

**Table 1:** Oligonucleotide primers used for real-time RT–PCR.

**Gene (Symbol)**	**Assay ID**	**Amplicon length**
Interleukin 1 beta (*IL-1β*)	Mm00434228_m1	90
Interleukin 6 (*IL-6*)	Mm00446191_m1	124
Chemokine (C-C) ligand 4 (*CCL4*)	Mm00443111_ml	70
Chemokine (C-C) ligand 7 (*CCL7*)	Mm00443113_ml	122
Chemokine receptor 3 (*CCR3*)	Mm01216172_m1	98
Chemokine receptor 5 (*CCR5*)	Mm01216171_ml	78
Interleukin 23 alpha (*IL-23α*)	Mm00518984_m1	61
Transforming growth factor beta 2 (*TGF-β2*)	Mm01321739_m1	109
Glyceraldehyde-3-phosphate dehydrogenase (*GAPDH*)	Mm99999915_gl	107

The assay ID is from Applied Biosystems.

## Results

### Experimental post-traumatic keratomycosis

All animals infected with *Candida albicans* strain SC5314 developed signs of keratitis by 24 h p.i. Mean±standard deviation (SD) disease severity scores of three independent *Candida albicans-*infected groups (five per group) were 8.0±0.7, 8.8±0.8, and 8.2±0.8. Based upon one-way ANOVA, no significant difference occurred among the three groups (p=0.27), and the overall mean ocular disease severity was 8.3±0.4. No clinical disease or obvious signs of infection was found in any eyes of the mock-infected control groups.

### Corneal gene expression

Triplicate samples of RNA from five-cornea pools of infected and control, mock-infected groups met criteria for microarray analysis (Table 2). Overall, the scaling factor was less than or equal to 3 or within two SD around the mean, and the average background level was less than 100 for all samples. All three probes were present for both housekeeping genes coding for β-actin and GAPDH, and as expected, two probes were present for the spike-in probe sets, BioB, BioC, BioD, and CreX. RNA degradation plots demonstrated similar patterns for all chips (data not shown).

**Table 2:** Results of RNA quality control evaluation.

**Experimental group**	**Scaling factor**	**Average background**	**PCall %**	***β-Actin* 3′to 5′ ratio**	***GAPDH* 3′to 5′ ratio**
Infected 1	2.5	64.5	56.9	2.6	2.7
Infected 2	2.5	54.1	54	2	2.1
Infected 3	2.4	55.8	52.4	1.8	1.9
Control 1	2.4	60.6	52.9	2.8	2.6
Control 2	2.4	54.9	53.7	3	2.6
Control 3	4.6	53.7	47.2	3.1	2.8

Triplicate samples of five-cornea pools were used from *Candida albicans*-infected or mock-infected control animals. PCall % is the percent of probe sets that are called “present” by Affymetrix’s algorithm. The 3′ to 5′ ratio is the 3′-end to 5′-end probe intensity ratio. The number of “present” probes for housekeeping probe sets (*β-actin* and *GAPDH*) was 3, and the number of spike-in probe sets (BioB, BioC, BioD, and CreX) was 2.

**Figure 1:**
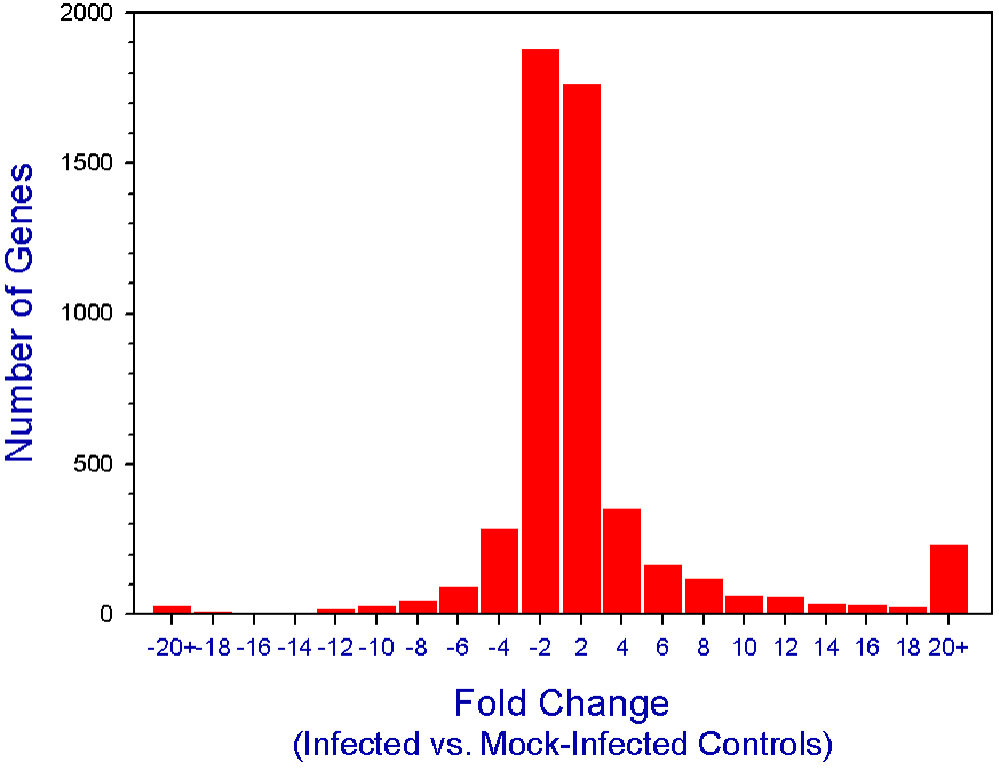
Distribution of differentially regulated corneal genes. The microarray results compare the number of corneal genes in mouse corneas from *Candida albicans*-infected to the number of corneal genes in mock-infected control mouse corneas. The results are categorized by expression-level differences in twofold increments. A minimum of significant (p≤0.05), twofold upregulation or significant (p≤0.05) twofold down-regulation was required to be charted.

Among the total of 45,102 genes detected by the Affymetrix Genechip 430.2 microarray, 3,977 genes (8.82%) in *Candida albicans-*infected corneas were significantly (p≤0.05) and differentially (≥2.0 fold change) regulated compared to mock-infected control corneas. Of these, 1,987 genes were upregulated, and 1,990 were down-regulated. With a criterion of at least 4.0 fold significant change in the expression level, 1,672 genes (3.71%) were differentially expressed with 1,075 upregulated and 597 down-regulated. Thirty different genes were significantly upregulated more than 100 fold (Table 3). The frequency of significant gene expression followed a bell-shaped distribution (Figure 1).

**Table 3:** Categories of all genes significantly upregulated more than 100 fold.

**Category**	**Symbol**	**Description**	**GenBank accession**	**Relative upregulation**	**p value**
Chemokines	*PPBP*	Chemokine (C-X-C) ligand 7	AB042817	834.4	0.01
	*CCL3*	chemokine (C-C) ligand 3	AA895994	374.3	0.04
	*CXCL3*	chemokine (C-X-C) ligand 3	AK144158	291.7	0.04
	*CCL4*	chemokine (C-C) ligand 4	AF128218	145.2	0.011
	*CXCL5*	chemokine (C-X-C) ligand 5	AK044397	132.4	0.05
Metalloproteinases	*MMP13*	matrix metallopeptidase 13	AK150728	375.4	0.001
	*ADAM8*	a disintegrin and metallopeptidase domain 8	AK089086	108	0.01
	*MMP8*	matrix metallopeptidase 8	AK089234	102.5	0.0003
Interleukin cytokines	*IL-6*	interleukin 6	AK089780	245.4	0.009
	*IL-1β*	interleukin 1 beta	AK156396	136.8	0.03
	*OSM*	oncostatin M	AK087945	122.7	0.006
Leukocyte chemotaxis	*FPR1*	formyl peptide receptor 1	AK137714	145.2	0.01
	*FPR-RS2*	formyl peptide receptor, related sequence 2	AF071180	132.8	0.05
	*SELL*	selectin, lymphocyte	AK137509	114.3	0.01
Leukocyte surface molecules	*CLEC4D*	C-type lectin domain family 4, member d	AF061272	118.2	0.04
Ig superfamily receptors	*CLEC4E*	C-type lectin domain family 4, member e	AB024717	180	0.02
	*TREM1*	triggering receptor expressed on myeloid cells 1	AF241219	136.4	0.0063
Cell proliferation molecules	*CSF3*	colony stimulating factor 3 (granulocyte)	AK145177	114.3	0.001
	*HMGA2*	high mobility group AT-hook 2	AC153362	106.8	0.002
	*G0S2*	G0/G1 switch gene 2	AK003165	104.7	0.01
Neuro-hormone mediators or receptors	*IRG1*	immunoresponsive gene 1	AK036446	328.9	0.03
	*NPPB*	natriuretic peptide precursor type B	AB039044	204.1	0.000002
	*MRGPRA2*	MAS-related GPR, member A2	AK156116	202.6	0.008
	*INHBA*	inhibin beta-A	AC154742	201.9	0.0003
Histamine synthesis	*HDC*	histidine decarboxylase	AB039880	251.5	0.03
Prostaglandin synthesis	*PTGS2*	prostaglandin-endoperoxide synthase 2	AC114655	106.2	0.004
Intracellular molecules	*CYP4F18*	cytochrome P450, family 4, subfamily f, polypeptide 18	AF233647	164.8	0.01
	*SAMSN1*	SAM domain, SH3 domain and nuclear localization signals, 1	AC166831	145.7	0.002
	*PLEK*	pleckstrin	AF073294	142.6	0.003
Protein transport	*SLC15A3*	solute carrier family 15, member 3	AF121080	114.2	0.02

**Figure 2:**
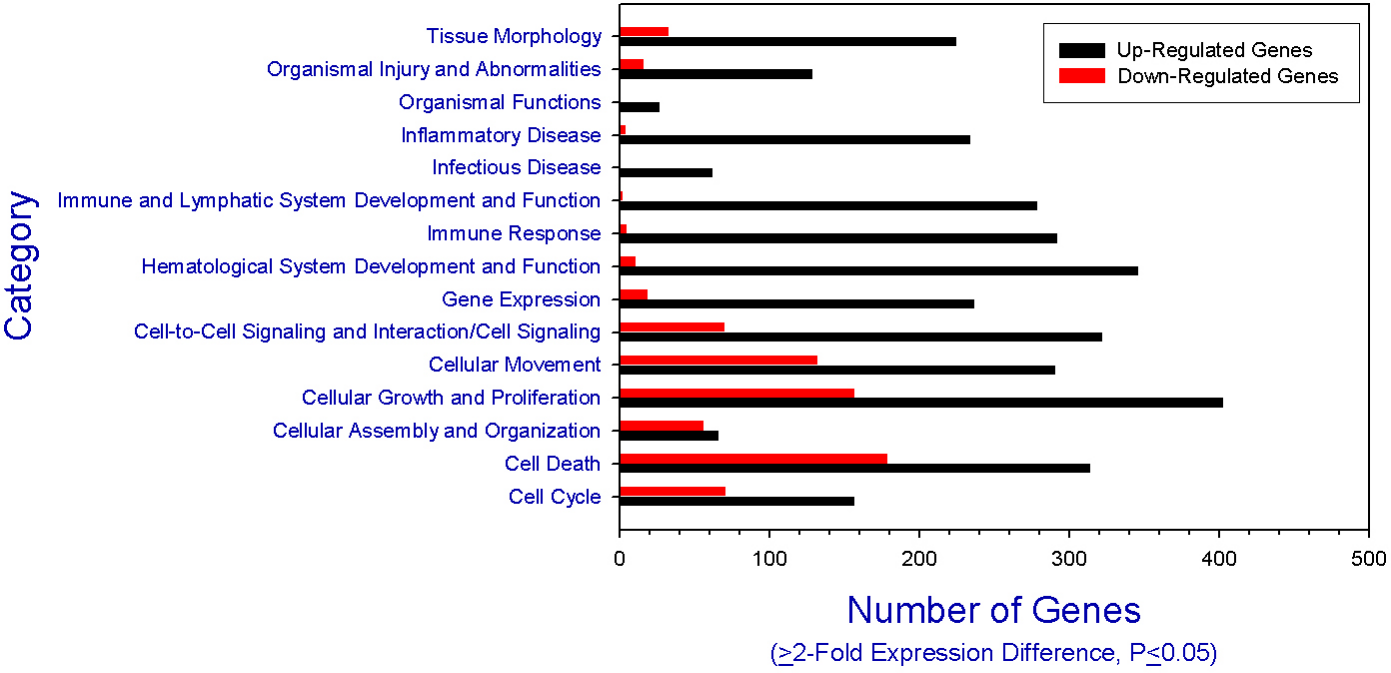
Differential upregulation and down-regulation of corneal genes. Microarray results compare gene expression levels in mouse corneas from *Candida albicans*-infected compared to corneas from mock-infected controls. The results were assigned to general categories based upon the known functions of the gene products. The number of genes expressing a twofold or greater significant difference (p≤0.05) is plotted for the listed categories. Red bars represent the number of genes significantly down-regulated, and black bars represent the number of genes significantly upregulated.

Gene expression levels were assigned to general categories based upon known functions of gene products and the number of genes expressing greater than or equal to twofold significant difference (p≤0.05). While functional categories included some down-regulated genes, the number of upregulated genes was consistently more prevalent (Figure 2). Canonical analysis showed that the ratio of significantly upregulated genes within the pathways ranged from 0.13 to 0.34 (Table 4). Several signaling pathways included genes that were upregulated fourfold or more (Appendix 1). Similar results were found for selected genes detected by microarray and real-time reverse transcriptase (RT)–PCR analysis (Table 5), although *CCR3* expression differed between the infected and mock-infected corneas.

**Table 4:** Upregulated pathways in *Candida albicans* keratitis compared with mock-infected controls.

**Signaling pathway**	**Log** **(p value)**	**Number of genes involved**	**Number upregulated genes**	**Ratio**
Leukocyte extravasation	16	191	44	0.23
NF-κβ	15.5	141	38	0.27
Interleukin 10	13.2	68	23	0.34
Interleukin 6	12.5	90	27	0.3
Acute-phase response	11.3	171	36	0.21
B cell receptor	6.7	144	26	0.18
Toll-like receptor	6.6	50	14	0.28
Integrin	6.1	188	30	0.16
Natural killer cell	6	112	19	0.17
Granulocyte-macrophage colony-stimulating factor	5.8	63	15	0.24
Transforming growth factor beta	4.9	84	16	0.19
Chemokine	4.7	75	15	0.2
Interleukin 2	4.5	52	12	0.23
Insulin-like growth factor 1	3.8	94	15	0.16
T cell receptor	3.5	100	15	0.15
Epidermal growth factor	3	47	9	0.19
Vascular endothelial growth factor	3	93	13	0.14
Interleukin 4	3	69	11	0.16
JAK/STAT	2.6	112	19	0.17
Apoptosis	2.5	62	8	0.13

**TABLE 5:** Real-time RT-PCR confirmation of microarray.

**Gene (Symbol)**	**Microarray**	**Real-time RT-PCR**	**p value**
Interleukin 1 beta *(IL-1β)*	342.4±477.5	378.0±317.4	0.92
Interleukin *6 (IL-6)*	376.0±295.4	388.1±318.9	0.96
Chemokine (C-C) ligand 4 (*CCL4)*	228.7±175.5	200.9±172.2	0.85
Chemokine (C-C) ligand 7 (*CCL7)*	60.9±51.7	14.9±9.8	0.21
Chemokine receptor 3 (*CCR3)*	-6.0±3.5	14.5±4.3	0.004
Chemokine receptor 5 (*CCR5)*	1.1±1.1	2.3±0.9	0.20
Interleukin 23 alpha *(IL-23α)*	9.1±3.3	14.2±9.9	0.44
Transforming growth factor beta 2 *(TGFβ2)*	1.8±1.0	-1.8±3.7	0.19

Mean fold change ± standard deviation, comparing infected to mock-inoculated control corneas.

## Discussion

Fungal keratitis comprises a dynamic interaction between microorganisms and host [16]. Because most mechanisms that mediate the pathogenesis of fungal keratitis have not been elucidated, we explored the global gene expression during *Candida albicans* corneal infection with the use of genetic microarray. To control for the effects of corneal trauma [17,18], we compared corneas having post-traumatic fungal keratitis with scarified controls. At 24 h p.i., approximately 9% of genes were significantly expressed twofold or more and nearly 4% were differentially altered at least fourfold.

Our findings complement previous studies of microbial keratitis. Huang and Hazlett [11] found that approximately 10% of genes changed significantly 24 h after the onset of *Pseudomonas aeruginosa* keratitis in BALB/c and B6 mice. Wang and colleagues [10] exposed heat-killed spores of *Aspergillus fumigatus* to excised pieces of BALB/c mouse cornea and noted that several host genes were substantially upregulated including those encoding interleukin cytokines such as IL-3. These gene expression studies imply that microbial keratitis involves synchronized host processes that affect several aspects of inflammatory and immune responses, intercellular communication, and cellular metabolism.

Toll-like receptors (TLRs) may carry out the initial recognition of fungi at the corneal surface [19]. After binding microbial pathogens [20,21], TLRs can induce intracellular cascades that give rise to inflammatory cytokines. Several members of the TLR family that are expressed on the cornea respond to fungal infection [22,23], and our preliminary analysis suggests that TLR pathways are involved in *Candida albicans* keratitis.

The recruitment of acute inflammatory cells is a key event that immediately follows fungal adherence. Among genes detected by microarray, a quarter of them are involved in signaling leukocyte extravasation, and these genes were significantly upregulated. *Candida albicans* infection triggers chemotactic and other signaling cascades [22] with the nuclear factor-ĸβ pathway having an important role. This transcription factor regulates immune responses to infection and is increased during keratitis [24].

Several mediators of inflammation and wound healing such as metalloproteinases and interleukins are active in the initial events of corneal infection. Genes encoding IL-6 and IL-1β were expressed more than 300 fold during fungal keratitis. Corneal injury results in the upregulation of IL-1β [18], and infection further increases its expression [25]. IL-6 and IL-1β activate neutrophils during *Candida albicans* infection [26]. By validating our microarray results for selected genes that had variable expression patterns, we showed that the early stage of fungal keratitis involves upregulation of proinflammatory interleukins in a pattern similar to that during bacterial keratitis [11]. We also confirmed that TGF-β does not appear to be altered during early fungal infection [27]. While injury induces interleukins and related cytokines, infection enhances the production of selected mediators of acute inflammation.

Our studies of fungal keratitis are consistent with other models of *Candida albicans* infection. Expression patterns of genes coding for IL-6 and chemokine receptors markedly increase soon after the onset of *Candida albicans* exposure [27], and this increase likely contributes to the recruitment and influx of leukocytes into the infected cornea. Histopathological evidence also indicates that corneal inflammation during the incipient stage of *Candida albicans* keratitis is a manifestation of the innate immune response [13].

In parallel with pathways promoting ulcerative keratitis, the cornea has anti-inflammatory mechanisms for inhibiting NF-ĸβ activity and dampening the effects of inflammatory mediators. Although increased during bacterial keratitis, IL-10 was not significantly altered 24 h after corneal inoculation with yeasts, possibly because of a different kinetic profile. However, approximately one-third of genes involved in IL-10 signaling were significantly upregulated during *Candida albicans* keratitis. Other inflammatory suppressors such as suppressor of cytokine signaling protein-3 were also upregulated.

Pathways of immune recognition may also be activated by fungal infection. We confirmed that IL-23 increases during *Candida albicans* keratitis [27]. Expression of this T-cell regulatory molecule suggests that adaptive immunity may be involved in candidiasis, although to a lesser degree than innate immune reactions [28].

Our study provides evidence for a likely scenario of molecular events during early fungal keratitis. Soon after corneal surface injury and exposure to viable fungi, fungal components are recognized by host receptors such as lectins and toll-like receptors. Binding by ligands induces intracellular cascades that activate NF-ĸβ, which, in turn, upregulates genes responsible for inflammatory responses. Corneal levels of interleukins and other cytokines rapidly increase. Neutrophils and macrophages are recruited toward the site of infection, helping to kill fungi but releasing chemokines that contribute to corneal ulceration and neovascularization.

In summary, gene microarray analysis is a useful means for understanding the molecular biology of ocular inflammatory disease. *Candida albicans* keratitis involves the induction of genes encoding several interrelated pathways, and this investigation provides insight into the pivotal reactions during the onset of fungal keratitis. Characterizing this coordinated sequence of events can provide insight into the pathogenesis of fungal keratitis that will open up new opportunities for therapeutic interventions.

## Appendix 1. Upregulated signaling pathways in *Candida albicans* keratitis compared with mock controls.

To access the data, click or select the words “Appendix 1”. This will initiate the download of a (pdf) archive that contains the file.
